# Exacerbation of Osteoarthritic Joint Pain by Lyme Disease

**DOI:** 10.7759/cureus.59318

**Published:** 2024-04-29

**Authors:** Athena Z Bennani, Brian Chegwidden, Constantino G Lambroussis, Lorrie Penfield

**Affiliations:** 1 College of Medicine, Lake Erie College of Osteopathic Medicine, Elmira, USA; 2 College of Medicine, Lake Erie College of Osteopathic Medicine, Elmira, NY, USA; 3 Osteopathic Medicine/Family Medicine, Lake Erie College of Osteopathic Medicine, Elmira, USA; 4 Internal Medicine/Medical Education, Arnot Ogden Medical Center/Lake Erie College of Osteopathic Medicine, Elmira, USA

**Keywords:** arthritis, osteoarthritic, borrelia, borreliosis, lyme

## Abstract

Lyme disease progresses through three distinct clinical phases: early localized, early disseminated, and late disseminated. Lyme arthritis is characterized by attacks of joint swelling lasting for weeks to months, potentially causing permanent joint damage in late disseminated disease. Our case focuses on a 63-year-old, obese, type 2 diabetic, wheelchair-bound, Caucasian male with severe bilateral knee pain. Our patient had previously undergone bilateral knee arthroscopies for meniscal tears and also had knee injections performed previously without the desired level of pain alleviation. He indicated a recent cough that was treated with erythromycin and noted his knees felt better during the course of the antibiotic. The patient recreationally enjoyed hunting and mentioned that his dog had Lyme Disease. Laboratory confirmation of Lyme disease prompted our patient to be treated with doxycycline. Upon completion of doxycycline therapy, our patient noted significant improvement in his knee pain. The improvement was significant enough that the patient canceled a planned bilateral knee replacement with his orthopedic surgeon, and no longer required the use of a wheelchair as he was able to return to ambulating independently. The patient’s quality of life improved significantly, and he could also return to work. Lyme disease should be a consideration in the differential diagnosis of patients in areas endemic to the disease, and patients who tend to have outdoor lifestyles.

## Introduction

Borreliosis, also known as Lyme disease, is a tick-borne illness most commonly caused by the spirochete Borrelia burgdorferi in North America [1]. There are roughly 300,000 new cases of Lyme each year in the United States, most of which occur in the Northeast [2]. Lyme disease progresses through three distinct clinical phases. Early localized disease involves the spreading of the spirochetes within the dermis at the site of the tick bite. The initial presentation includes erythema migrans, which occur in 80% of patients, with fever, lymphadenopathy, and other flu-like symptoms [3,4]. Several months after the initial tick bite, the disease progresses to early disseminated disease, when the spirochetes spread hematogenously. Common yet serious signs of early disseminated disease include cardiac symptoms, such as atrioventricular heart block and myopericarditis [5,6], neurologic symptoms including migratory arthralgias and mononeuropathy multiplex [6,7], along with musculoskeletal symptoms such as intermittent or persistent arthritis in one or multiple large joints, especially the knee [4,5]. Finally, late disseminated disease develops, causing chronic arthritis with severe joint damage and polyneuropathy. B. burgdorferi strains disperse into joints, tendons, or bursae as infection progresses [8]. Lyme arthritis is characterized by attacks of joint swelling lasting for weeks to months, potentially causing permanent joint damage in late disseminated disease [9]. This can worsen the symptoms of patients already suffering from joint conditions such as rheumatoid arthritis and osteoarthritis.

It is believed that a majority of the pathogenesis caused by B. burgdorferi is caused by the immune response and inflammation due to bacterial infiltration since it does not produce endo- or exotoxins that damage the host. Instead, it continuously evades the host’s immune system through antigenic variation [4] and evasion of complement-mediated killing [10]. B. burgdorferi possesses multiple coding sequences for the antigenic surface protein, VIsE, which undergoes recombination once the host starts to produce antibodies against it, resulting in the host having to produce entirely new antibodies against the new surface protein [10,11].

Additionally, B. burgdorferi evades the complement pathway by producing outer surface protein E-related proteins (Erps) and complement regulator-acquiring surface proteins (CRASPs), which bind to complement factor H and complement factor H-like protein [12,13]. Factor H and factor H-like proteins then bind and inactivate C3b and prevent the spirochete from complement-mediated killing [10]. This allows the spirochetes to continue to infiltrate and multiply in tissues all over the body, which further increases the activation of immune effector cells and the release of cytokines and pyrogens, causing fever, myalgias, and arthralgias [10]. Persistent inflammation within the joint space eventually leads to Lyme arthritis, which closely resembles rheumatoid arthritis. It presents with villous hypertrophy, lining-cell hyperplasia, and abundant lymphocytes and plasma cells within the synovium [4].

Treatment of Lyme disease involves antibiotic therapy, usually a 28-day course of doxycycline, amoxicillin, and cefuroxime. Nonsteroidal anti-inflammatory drug (NSAID) therapy may also be beneficial to patients with painful Lyme arthritis to reduce joint inflammation [9].

## Case presentation

**Figure 1:**
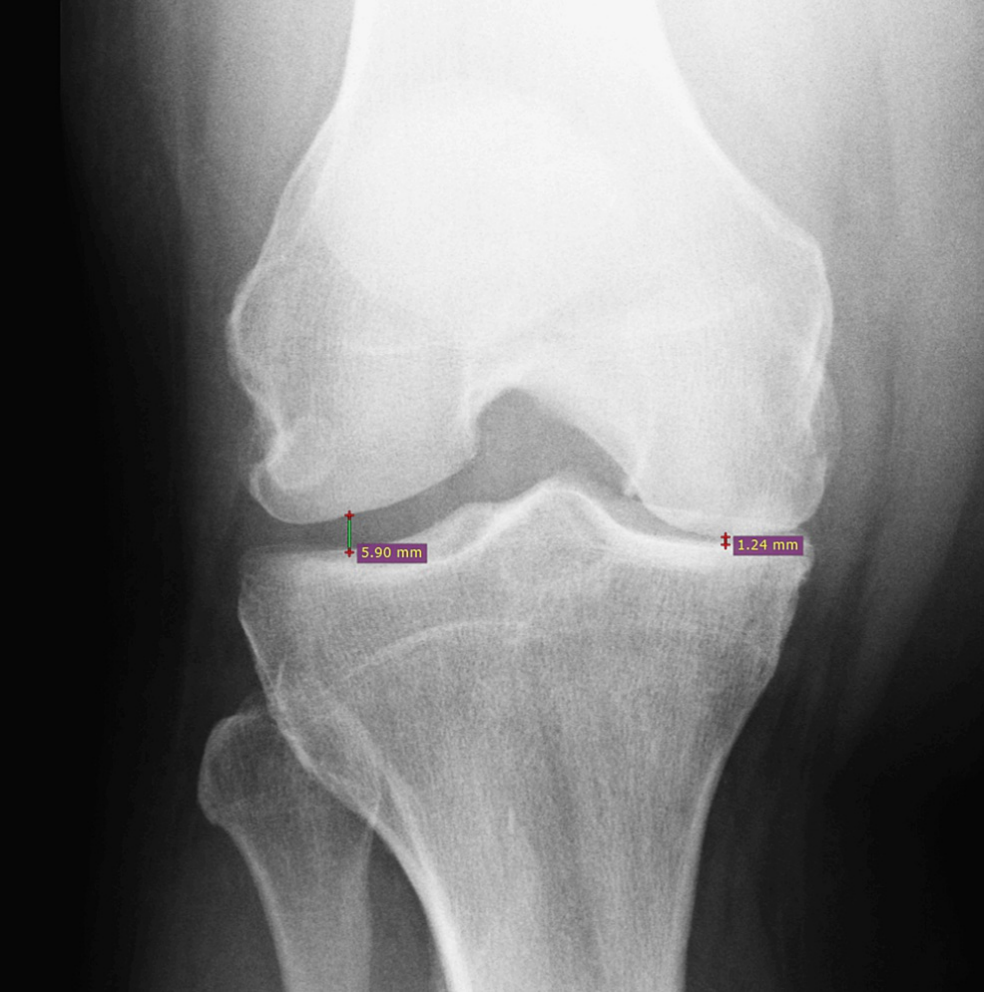
Right knee PA view. Significant joint space narrowing of the patient's right knee, estimated at 1.24 mm on the medial aspect. The lateral aspect of the knee shows an estimated 5.90 mm joint space. PA, posterior-anterior

**Figure 2:**
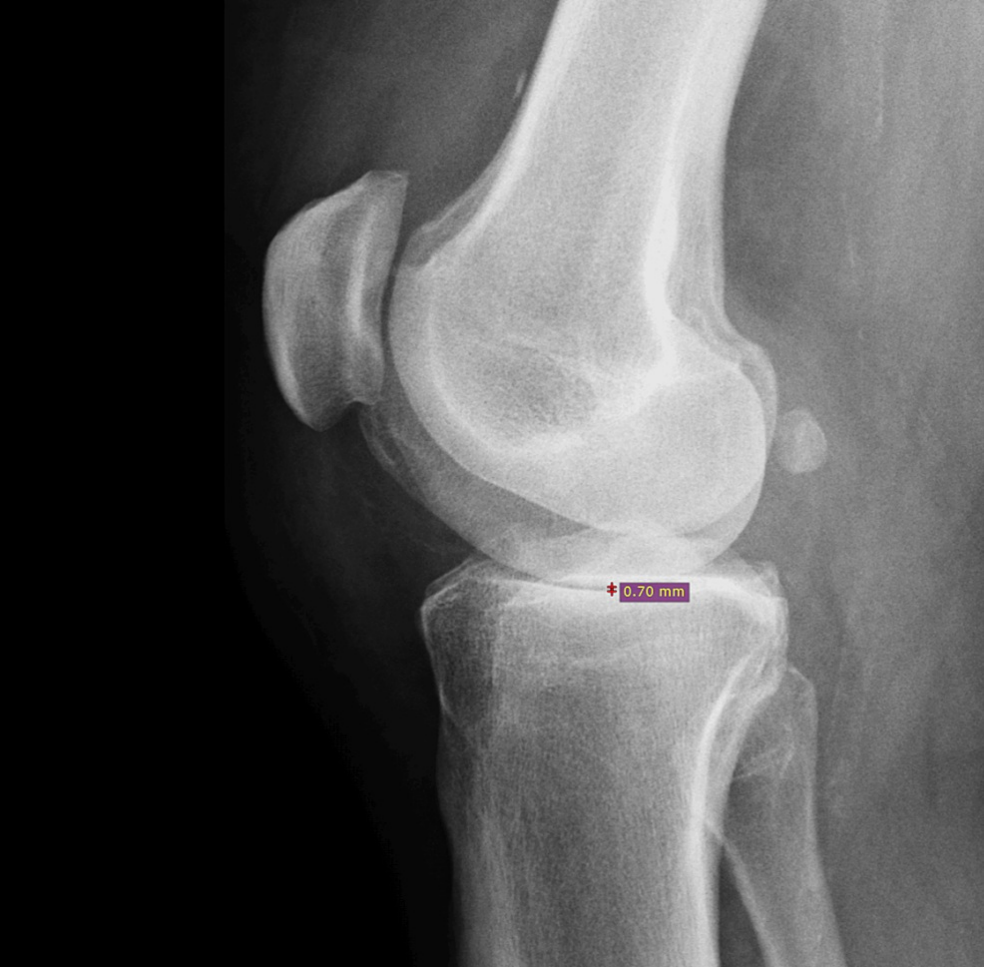
Lateral erect view of the right knee. Joint space narrowing, space estimated at 0.70 mm.

Our patient was a 63-year-old Caucasian male, nonsmoker, and type 2 diabetic, who presented to the office with severe bilateral knee pain progressively worsening over the past several months. The patient rated the pain as a persistent baseline of 4 out of 10, with elevations in pain with activity. The patient’s most recent hemoglobin A1C was 7.8. The patient indicated that he had recently undergone an ultrasound of the lower extremity secondary to calf pain; however, the results of that study indicated zero evidence of a deep vein thrombosis (DVT). This ultrasound study was performed secondary to patient symptomatology as well as an elevated D-dimer level. Effusions were noted to the knee joints bilaterally. Orthopedic surgery had also been consulted after DVT was ruled out, and the patient was being seen by them for a planned bilateral knee replacement. He had previously undergone bilateral knee arthroscopies for meniscal tears. The patient additionally had corticosteroid knee injections performed previously; however, these did not provide the desired pain alleviation. The patient’s most recent knee radiographs are shown in Figures 1-2, both showing joint space narrowing consistent with osteoarthritis of the knee.

The patient's past medical history consisted of diabetes mellitus type 2 (Type 2 DM), hypertension, hyperlipidemia, coronary artery disease (CAD), obesity (body mass index [BMI] 40.72), dyspepsia, lactose disaccharidase deficiency, angioedema, and benign neoplasm of the colon. The patient’s surgical history consisted of an appendectomy, bilateral knee arthroscopies for meniscal tears, cardiac catheterization, and polypectomy. The patient’s family history included the mother having had cardiac valve replacements as well as colon cancer. The patient’s father had diabetes mellitus, as well as coronary artery disease that required quadruple bypass. The patient’s younger brother also had a history of colon cancer. The patient did not consume alcohol in excessive amounts or utilize any illicit substances. He was married, had three children consisting of one son and two daughters, and worked as a chief executive officer (CEO) of a company.

The patient did report that he was recently on erythromycin for an upper respiratory infection (URI) and indicated that his knees felt better while he was taking erythromycin. The patient did indicate that he hunted regularly and that his dog had Lyme disease. Fluid from the knee effusions was obtained and sent for laboratory analysis. The results indicated no organismal growth, and no crystals were noted under microscopy. As the patient’s dog had Lyme disease, Lyme IgG and Lyme IgM studies were ordered, both of which resulted above the normal reference range of 0.00 to 0.79. The lab’s equivocal reference range was 0.80 to 1.20, and the positive range was any value greater than 1.20. The patient’s Lyme IgG was reported as 4.0 and Lyme IgM was reported as 2.2. Lyme disease immunoblot revealed IgG bands of p18, p28, p39, p41, p66, and p93. Additionally, p39 IgM bands were also found in the immunoblot. 

With the presence of six bands of IgG noted on the immunoblot, this confirmed the diagnosis of Lyme disease in our patient. Upon laboratory confirmation of the diagnosis of Lyme disease, the patient was treated with doxycycline. Upon completion of doxycycline therapy, the patient additionally noted significant improvement in his bilateral knee pain. The improvement was noted to be significant enough that the patient canceled the planned bilateral knee replacement with the orthopedic surgeon. 

## Discussion

Before treatment, our patient was wheelchair-bound due to the combination of existing osteoarthritis and the manifestation of Lyme disease in his knees. His joint space width was estimated at 1.24 mm on the medial aspect and 5.9 mm on the lateral aspect of the knee. Normal knee joint space width measurements for healthy individuals are typically near 5.7 mm medially and laterally for males and near 4.8 mm for females comparatively [14]. He received bilateral knee injections, which failed to alleviate his symptoms. This failure to alleviate symptoms after the injections could be attributed to decreased immune response to the area of infection. While corticosteroids prompt neutrophilic leukocytosis, there has been speculation that corticosteroids alter the adhesion molecules (E-selectin and ICAM-1) of neutrophils and therefore prevent them from reaching the inflammatory site, the knee joint. Additionally, there was a noticeable decrease in other circulating white blood cells. Specifically, a decrease in lymphocytes means that there are fewer antibodies (B lymphocytes) and killer T cells (T lymphocytes) circulating and eradicating the bacterial infiltration within the knees. This impact is caused by a downregulation of lymphocyte adhesion molecules, LFA-1 and CD-2, by action of the corticosteroid receptors [15]. With the steroids effectively preventing inflammation from occurring at the joint by downregulating adhesion molecules for white blood cells, the bacterial infection can proliferate at the sites of infection and continue to destroy the tissues of the knees, while the patient may experience temporary relief of pain symptoms.

The impact of steroid injections on the patient’s diabetic control and their potential contribution to the proliferation of infection already occurring are additional factors to consider in our patient. Upon the patient’s presentation, there was a reported last glycosylated hemoglobin value of 7.8 or an average blood glucose level of 154-183 [16]. With the addition of a steroid medication being used for this patient’s pain management, the steroid further led to an elevation of the patient’s glucose levels. The stress of infection on the patient’s body also contributed to an increase in our patient’s basal glucose levels. With the elevated glucose levels persisting, it could be speculated that there is a greater environment for bacterial infection to proliferate. Furthermore, the patient’s elevated glucose levels could lead to additional complications involving his history of CAD and hyperlipidemia by promoting further development of atherosclerotic lesions systemically, particularly in his coronary arteries.

The erythromycin used for the patient’s cough helped alleviate some symptomatic knee pain for our patient. Macrolides have been shown to combat late disseminated Lyme disease infection, although they are not recommended as a first-line therapy [17]. The patient would have been on a short course of the antibiotic, but not long enough to eliminate the entire infection.

Cefazolin, a first-generation cephalosporin, when given as the antibiotic for preoperative prophylaxis, has a greater chance of preventing prosthetic joint infection in comparison to other antibiotics specifically for total knee and total hip arthroplasties [18]. If the patient has a known methicillin-resistant Staphylococcus aureus (MRSA) history, the prophylactic antibiotics would be modified to include vancomycin. In light of this, standard precautions for antibiotic prophylaxis of the patient for joint replacement would not have any beneficial impact on the current infection within the joint and instead would eliminate other microbes within the joint. This would result in the cessation of any competitive inhibition of proliferation of the Lyme disease.

The patient’s comorbidities of type 2 DM, CAD, and obesity would have put them at an increased risk for localized complications, including periprosthetic joint infection, surgical site infection, dislocation of the new joint, as well as elevated strain on the knees and surrounding structures. Furthermore, the patient is at greater risk for systemic complications such as coronary artery disease, acute kidney injury, cardiac arrest, deep vein thrombosis, and pulmonary embolism [19,20]. Considering our patient is already in a hypercoagulable state due to CAD, his chances of developing one of the aforementioned systemic complications increase as much as 40% to 80% per surgery [21].

The expected length of hospital stay for our patient would be estimated from 3 to 10 days depending on extenuating circumstances, which include the expectation of the patient, surgeons, physiotherapists, occupational therapists, and nurses; hospital transport; delays in supplying aids to the patient's homes; and the prescription of discharge medication not being completed in good time. It should be further noted that early mobilization of the joint allows for quicker recovery time due to the prevention of fibrotic changes in the joint due to the active movement, along with a boost in the patient’s confidence upon seeing that the surgery was successful. After being discharged from inpatient care utilizing the accelerated rehabilitation program, the patient could be expected to undergo six weeks of physical therapy to return to their normal functional level [22]. These speculations are most likely made with unilateral knee replacements in mind and might not include bilateral or simultaneous knee replacement. Under these circumstances, the patient would likely have to go through this process twice, making the full recovery after 12 weeks of rehabilitation, six weeks for each knee assuming the second surgery was completed right after the patient was cleared for full activity from the first surgery [22]. Our patient would likely take a longer amount of time to recover secondary to rehabilitating both knees, one after another, due to comorbidities impacting functional ability, including type 2 DM, CAD, obesity, hyperlipidemia, and being in a hypercoagulable state secondary to the surgeries predisposing the patient to potentially lethal clot formation. With these factors in mind, the patient’s recovery time would be 12+ weeks, with both knees being replaced and no extenuating circumstances interrupting the rehabilitation protocol and timeline. In conjunction with rehabilitation time, the patient would have to miss work for his recovery, impacting his daily activities and company involvement, unless he was able to work remotely while being inpatient and attending physical therapy. His company would likely have suffered from the absence of its CEO for approximately three months, as they waited for the patient to recover from both surgeries. This aspect would demonstrate a social factor to the patient’s stressors about moving forward with the surgery and rehabilitation.

## Conclusions

After completion of a 28-day course of doxycycline, our patient regained his ability to walk independently and canceled his knee replacements with orthopedic surgery. In addition to returning to work, the patient’s quality of life significantly improved as a result of his regained mobility. Our patient was able to avoid a costly, high-risk surgical procedure with the detection and treatment of his Lyme disease. Lyme disease should always be a consideration in the differential diagnosis of patients who have lived or have traveled to areas that are endemic to the disease and who tend to have outdoor lifestyles.
